# PepGM: a probabilistic graphical model for taxonomic inference of viral proteome samples with associated confidence scores

**DOI:** 10.1093/bioinformatics/btad289

**Published:** 2023-05-02

**Authors:** Tanja Holstein, Franziska Kistner, Lennart Martens, Thilo Muth

**Affiliations:** Section S.3 eScience, Federal Institute for Materials Research and Testing (BAM), 12205, Berlin, Germany; VIB-Ugent Center for Medical Biotechnology, 9052, Zwijnaarde, Belgium; Department of Biomolecular Medicine, Ghent University, 9000, Ghent, Belgium; Section S.3 eScience, Federal Institute for Materials Research and Testing (BAM), 12205, Berlin, Germany; VIB-Ugent Center for Medical Biotechnology, 9052, Zwijnaarde, Belgium; Department of Biomolecular Medicine, Ghent University, 9000, Ghent, Belgium; Section S.3 eScience, Federal Institute for Materials Research and Testing (BAM), 12205, Berlin, Germany

## Abstract

**Motivation:**

Inferring taxonomy in mass spectrometry-based shotgun proteomics is a complex task. In multi-species or viral samples of unknown taxonomic origin, the presence of proteins and corresponding taxa must be inferred from a list of identified peptides, which is often complicated by protein homology: many proteins do not only share peptides within a taxon but also between taxa. However, the correct taxonomic inference is crucial when identifying different viral strains with high-sequence homology—considering, e.g., the different epidemiological characteristics of the various strains of severe acute respiratory syndrome-related coronavirus-2. Additionally, many viruses mutate frequently, further complicating the correct identification of viral proteomic samples.

**Results:**

We present PepGM, a probabilistic graphical model for the taxonomic assignment of virus proteomic samples with strain-level resolution and associated confidence scores. PepGM combines the results of a standard proteomic database search algorithm with belief propagation to calculate the marginal distributions, and thus confidence scores, for potential taxonomic assignments. We demonstrate the performance of PepGM using several publicly available virus proteomic datasets, showing its strain-level resolution performance. In two out of eight cases, the taxonomic assignments were only correct on the species level, which PepGM clearly indicates by lower confidence scores.

**Availability and implementation:**

PepGM is written in Python and embedded into a Snakemake workflow. It is available at https://github.com/BAMeScience/PepGM.

## 1 Introduction

Viruses, and especially viral pathogens, represent a tremendous threat to public health. While the threat of emerging viral diseases has been known to the scientific community for the past several decades (Morse 1997, Lipkin and Firth 2013, Peckham 2020), the severe acute respiratory syndrome-related coronavirus (SARS-CoV)-2 pandemic has propelled the importance of viral surveillance and investigation to the forefront of public attention (Riley et al. 2021, Hirabara et al. 2022). In this context, an essential element is the fast and accurate taxonomic inference of viral samples. As different strains of the same viral species can differ strongly regarding the patient outcome and epidemiological characteristics (Gussow et al. 2020, Haddad et al. 2021, Hirabara et al. 2022, Hu et al. 2022), correct strain-level attribution is crucial. While the gold standard method for taxonomic inference of viral samples is based on genomic analysis by NGS (Jones et al. 2017, Brown et al. 2018), bottom-up proteomics-based analysis is a promising orthogonal approach (Grossegesse et al. 2020). The public need for alternative bioanalytical tools is stressed by reagent shortages during global pandemic situations due to only a few available analysis methods (Smith et al. 2020, Chu et al. 2020).

The correct strain-level taxonomic inference of proteomic samples, however, remains a challenging task. An experimental approach that has been pursued in the past is the use of MALDI-TOF for the so-called biotyping or proteotyping (Sandrin et al. 2013, Singhal et al. 2015, Boyer et al. 2017). These approaches rely on signature peptides that are detected by mass spectrometry and uniquely associated with a species. Yet these approaches are limited to a single MS level and tend to be inappropriate for strain-level analysis, as tailored spectral databases with the strain-level resolution are required (Singhal et al. 2015). Thus, approaches based on tandem mass spectrometry (MS/MS), that offer a more precise view of the actual peptide sequence, have become a promising alternative (Gekenidis et al. 2014). For instance, an MS/MS-based approach for the identification of SARS-CoV-2 from patient samples was recently developed (Van Puyvelde et al. 2021) based on a targeted proteomics workflow.

For untargeted identification of viral samples based on MS2 spectra, only a few bioinformatic workflows exist (Penzlin et al. 2014, Alves et al. 2016, Mesuere et al. 2018, Kuhring et al. 2020) and these focus on the analysis of samples with known organism source (Aslam et al. 2017). For the identification of virus strains of unknown taxonomic origin, specific challenges emerge. Many of these overlap with challenges in metaproteomics, a field that aims to identify and quantify the phenotype of microbial communities with unknown taxonomic composition (Wilmes et al. 2015).

First, the analysis of samples with unknown composition potentially requires large search spaces. This is because standard proteomics workflows rely on search algorithms that aim to match experimental MS/MS spectra against a reference database (Verheggen et al. 2020). Thus, all candidate sequences have to be included in the reference database, which is computationally expensive and increases the risk of false positives (Muth et al. 2015). Second, unique protein inference is not guaranteed. A single peptide can map to several proteins, impeding accurate protein inference and leading to the so-called protein inference problem (Nesvizhskii and Aebersold 2005, Huang et al. 2012). Recently, graphical models have been applied to address this (Pfeuffer et al. 2020). Third, taxonomic inference has an analogous issue to protein inference: peptides are shared not only between proteins but also between taxa, impeding accurate taxonomic inference.

Specific bioinformatic workflows that were developed to address some of the described issues employ different strategies with varying success rates. Originally developed for metaproteomics, Pipasic relies on abundance similarity correction to taxonomically resolve samples with multiple closely related strains (Penzlin et al. 2014). Similarly, MiCId uses peptidome similarity correction (Alves et al. 2016) to achieve species-level resolution and provide a statistical estimate of its taxonomic assignments; however, it lacks strain-level resolution and has difficulties processing very large databases (Kuhring et al. 2020). Other tools originally developed for metaproteomics, such as Unipept (Mesuere et al. 2018), map peptides to their lowest common ancestor—meaning the lowest taxonomic level they are specific to. This results in taxonomic inference without confidence estimates and is inappropriate when strain-level resolution is required, as can be the case in clinical settings, where disease severity and therapeutic decisions strongly depend on strain information. A different approach, TaxIt (Kuhring et al. 2020), reduces the search space while still taking into account as many reference proteomes as possible through an iterative approach that uses multiple identification steps (Kuhring et al. 2020). Among all mentioned workflows, it is most apt at achieving strain-level resolution but lacks confidence estimates for its taxonomic assignments.

We here present PepGM, a graphical model-based bioinformatic pipeline for taxonomic inference of viral proteome samples. Our approach results in the accurate detection of viral pathogens in proteome samples with strain-level resolution. In particular, PepGM scores and ranks taxonomic assignments, thereby providing statistical confidence scores. This becomes particularly relevant in situations of ambiguous or erroneous identifications, e.g., when the correct viral strain is not present in the database or when the available strain reference proteomes overlap heavily. PepGM starts with high-confidence peptide identifications of a standard database search against a generic viral reference database. Based on the resulting matches, all candidate taxa on strain level are inferred using weighted peptide-spectrum matches (PSMs). For these taxa, each available strain-level proteome is retrieved. These candidate peptide sequences and the taxa themselves are represented as nodes in a probabilistic graphical model (Koller and Friedman 2009). An edge between a taxon and a peptide is drawn if the respective candidate peptide is validated by the database search results. Using the loopy belief propagation algorithm, i.e., an algorithm for approximate Bayesian inference, which has been successfully applied for protein inference (Pfeuffer et al. 2020), PepGM computes the marginal distribution of taxa, which corresponds to the probability of their presence in the analyzed sample. We demonstrate that PepGM consistently identifies the correct viral strains on an exemplary set of viral proteomic samples from publicly available data, investigating various use cases. We show that the taxon posterior probabilities represent suitable confidence estimates of our newly developed method. The combination of strain-level resolution with confidence estimates makes PepGM particularly useful for applications in a clinical context, where therapeutic decisions require both very detailed and very confident identifications of pathogens.

## 2 Materials and methods

In the following section, we describe PepGM in detail. We start with an overview of the PepGM workflow, then define the individual steps, input and outputs, and provide a brief description of the graphical model and inference algorithm used.

### 2.1 Workflow overview

**Figure 1.**
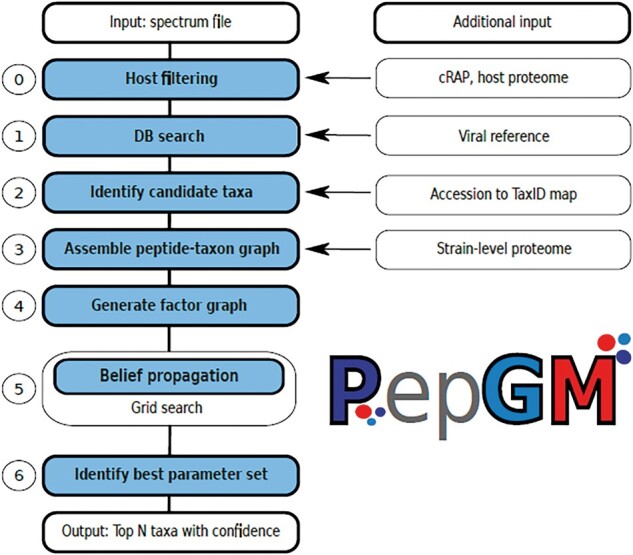
Schematic PepGM workflow for taxonomic inference of viral samples. The PepGM workflow comprises seven steps (0–6). The input is a raw MS/MS spectrum file in mgf format, additional input or information used at individual workflow steps are specified to the right of the workflow diagram.

PepGM takes as input MS/MS spectrum files (mgf format), a reference database containing one or multiple proteomes, and, optionally, a cRAP (common Repository of Adventitious Proteins containing common contaminants) and a host proteome database. PepGM broadly consists of the following seven steps (Fig. 1):

(0) optional host filtering, (1) standard proteomic database search, (2) identification of candidate taxa, (3) construction of the bipartite peptide-taxon graph, (4) construction of the graphical model, (5) inference algorithm embedded into a grid search, and (6) parameter evaluation and finally, results output and visualization.

We use Snakemake (Mölder et al. 2021) as a workflow management system, while all individual scripts are written in Python 10, developed and tested for Linux OS. Additional packages can be found in Supplementary Materials Section S1. We will now describe the individual workflow steps.

#### 0: Prefiltering (optional)

If a host and/or contaminant (e.g., cRAP) database is provided by the user, the MS/MS spectra are searched against these using SearchGUI (Barsnes and Vaudel 2018) for database-driven peptide identification and PeptideShaker (Vaudel et al. 2015) as a post-processing tool. The resulting matches are known to be of non-viral origin and can therefore be excluded from the input.

#### 1: Database search against a general viral reference database

Experimental spectra are searched against a complete virus-specific reference proteome database. This can be any database provided by the user in fasta format. However, due to the TaxID mapping in the following workflow step, proteins need to be provided with National Center for Biotechnology Information (NCBI) accession numbers. A TaxID is a unique identification number assigned to each taxon by NCBI, and a protein accession is a unique identifier for a protein. In this work, we use the RefSeq Viral database as a reference (O’Leary et al. 2016). As for the optional prefiltering, PepGM uses SearchGUI (Barsnes and Vaudel 2018) for database-driven peptide identification and PeptideShaker (Vaudel et al. 2015) as a post-processing tool. Due to its flexible Snakemake implementation, any combination of database search and rescoring algorithm is feasible. This step yields a list of scored peptides with corresponding PSMs. The identification confidence reported by PeptideShaker is the input to the taxon inference algorithm.

#### 2: Inferring candidate taxa

Based on these PSMs, we infer all candidate taxa and filter for high-scoring taxa as assessed by the following scoring scheme. The key idea is to score taxa based on their weighted PSMs per protein and, finally, per taxon. By filtering candidate taxa, we reduce the level of noise that the graphical model will be built on. PSM weights are first aggregated by summing up their individual scores at the protein level. The weight of a PSM is scaled by dividing it by the number of occurrences in other proteins, i.e., its degeneracy. Eventually, the aggregated weights are propagated in the same fashion to the candidate taxa. Taxa with a score above the global median score are considered high scoring.

#### 3: Construction of the bipartite peptide–taxon graph

Next, we assemble a bipartite graph of peptide and taxon nodes, where an edge is drawn between a taxon node and a peptide node if the peptide is part of an *in silico* trypsin-digested taxon proteome. The high-scoring candidate taxa (step 2) are taxonomic nodes at the species level, as this is the resolution provided by the NCBI taxon–protein mapping accessions. To gain access to strain-level information, all corresponding strain-level taxa are inferred from the species-level candidate taxa and kept as candidate strains. Each candidate strain proteome is automatically downloaded through the NCBI Entrez API and is *in silico* digested using tryptic cleavage settings. For now, trypsin is the only supported enzyme. The digested strain-level candidate peptides are matched (using exact string matching) against the set of peptides that were identified in the database search (step 1). Each match is included in the bipartite graph. Finally, the PSM scores are maximum aggregated at the peptide level.

#### 4: Construction of the factor graph

The subsequent Bayesian network representation that we choose for the joint distribution of peptides and taxa corresponds to a representation that has been successfully used for protein inference in previous work (Pfeuffer et al. 2020). The bipartite peptide–taxon graph represents the conditional dependencies between peptides and their parent taxa. Thus, the high-dimensional joint distribution of peptides and taxa is factorized into less complex distributions, which are the prior distributions of the taxa and conditional probability distributions (CPDs) for the peptides given the presence of a parent taxon. Both peptides and taxa are binary variables. As the actual CPD of the peptides depending on the presence of the taxa is not known, it is modeled using the noisy OR model (Díez and Druzdzel 2000). As for the protein inference model, this means that we assume that the presence of any of the parent taxa is sufficient to produce a peptide belonging to a taxon proteome. Three parameters, which we assume to be the same for all peptides and taxa, result from this model:

-
α : probability for a peptide to be observed given the presence of its parent taxon;
-
β : probability for a peptide to be randomly observed; and
-
γ : prior probability for a taxon to be present.

The following equation describes the probability for a peptide to be present (P=1) or absent (P=0), given a number N=n of parent taxa:


(1) $$\begin{array}{cc} p(P=0|N=n) & ={\left(1-\alpha \right)}^{n}(1-\beta )\\ p(P=1|N=n) & =1-p(P=0|N=n) \end{array}$$

Albeit this strong simplification, it has already proven useful to assume that α, β, and γ are equal for all peptides for protein inference (Pfeuffer et al. 2020). Following Equation (1), a CPD is computed for each peptide. Additional convolution tree (Serang 2014) nodes speed up the propagation of probabilities between peptide and taxon levels during the ensuing inference algorithm.

#### 5: Inference algorithm and grid search

Using the loopy belief propagation algorithm, posterior probabilities are calculated for all taxa in the graph. Loopy belief propagation iteratively updates the nodes (representing the variables—peptides and taxa) in the graph by passing messages between variable nodes. A message from one variable node to another represents the current estimate of the probability distribution over a variable based on the previous messages that it has received. Passing messages between variables is repeated until converged (Pearl 1988). The loopy belief propagation is embedded in a grid search through the parameter space of α, β, and γ. Each parameter α,β,γ∈[0,1]. The grid search covers a set of 126 parameter combinations with individual parameters being logarithmically spaced. Each parameter set provides a list of potential taxonomic inference results. By using Snakemake, the grid search can be parallelized easily, running on the number of cores specified by the user.

#### 6: Parameter evaluation and results output

With an empirically determined metric that exploits the distribution of taxon scores, we identify the parameter set that fits best for the sample at hand. The metric to be maximized is the following:


(2) $$M=\frac{1}{S_{1-10}}\times \frac{s_{1-3}}{s_{4-8}}\times \frac{1}{d_{1,2}^{2}+d_{2,3}+d_{3,4}}\times \frac{W_{1}}{W_{\max}}$$

In Equation (2), the subscripts have the following meaning: $S_{i-j}$ is the entropy of the distribution of the *i*-st to *j*-th posterior taxon probabilities. Including the entropy, which for a probability distribution *X* is defined as $S(X)=-\sum_{x\in X}p(x)\;\text{log}\;p(x)$ ensures that the distribution of the results selected contains as much information as possible. $s_{i-j}$ is the sum of the *i*-th to *j*-th posterior taxon probabilities, favoring distributions where the higher-scoring taxa clearly delimit themselves from the lower-scoring ones. $d_{i,j}$ is the taxonomic distance (calculated as the number of taxonomic nodes between two taxa) between the *i*-th and *j*-th taxa, the corresponding term favors distributions where the top-scoring taxa are taxonomically close. Finally, $W_{i}$ is the weight attributed to taxon *i* (or the parent species of taxon *i*) during the acquisition of candidate taxa, while $W_{\max}$ is the maximum weight any taxon was attributed during the analysis. This ensures that the top-scoring taxon identified by PepGM is also the one that was attributed to many PSMs. The inferred taxa plus their confidence scores are summarized as a simple bar plot, as a table, and as a visual projection onto a phylogenetic tree.

### 2.2 Selection of samples for evaluation and further parameter settings

To evaluate PepGM’s accuracy, we analyze several pathogenic viral samples that are publicly available in the PRIDE repository (Perez-Riverol et al. 2022). We have chosen the following samples: two *Cowpox virus (strain Brighton Red)* (PXD014913 and PXD003013), one *Human adenovirus 2* (PXD004095), two *SARS-CoV-2* (PXD024130 and PXD018594), one *Human herpesvirus 1 (strain 17)* (PXD005104), one *Hendra virus (strain Horse/Australia/Hendra/1994)* (PXD001165), and one avian bronchitis (Beaudette CK) (PXD002936). Throughout the article, we will refer to them as Cowpox virus PXD014923 or PXD003013, adenovirus, SARS-CoV-2 PXD024130 or PXD018594, herpesvirus, Hendra virus, and avian bronchitis, respectively. Details on the sample acquisition and sample-specific search parameter settings can be found in the Supplementary Materials. Except for SARS-CoV-2, all sample taxa were available on strain-level resolution in the NCBI taxonomy database. The host proteomes were downloaded using Uniprot.

In this study, we performed a database search using SearchGUI (X! Tandem search engine) and employed the RefSeq Viral reference database as our data source. This comprehensive database is maintained by the NCBI and can be accessed through their FTP server. The FDR (false discovery rate) was set to 5%, which is high compared to standard proteomic protocols. To assess the impact of incorporating a larger number of low-confidence PSMs, we conducted additional sample analyses using both 1% and 20% FDRs. Our findings revealed that the influence of varying FDRs on the results was minimal. To ensure an accurate assessment of PepGM’s performance, we examined the presence of specific strains used for evaluation in the NCBI RefSeq Viral database, as their presence might lead to an overestimation of our method’s effectiveness. We found that, with the exception of the human adenovirus 2 sample, these strains were not present in the database. Except for SARS-CoV-2, all of the selected samples previously served as benchmarking samples for the TaxIt (Kuhring et al. 2020) pipeline, where the taxonomic inference was benchmarked using MiCiD (Alves et al. 2016), Unipept (Mesuere et al. 2018), and Pipasic (Penzlin et al. 2014). It was shown that TaxIt performed best among all software, being the only one to consistently provide strain-level taxonomic inference, therefore, we compare PepGM directly to TaxIt. Run time and memory usage are evaluated using a Fujitsu laptop with Ubuntu 20.04.4 LTS, with a 4-core Intel i5-7200 CPU@2.50 GHz and 16 GB of memory.

## 3 Results

### 3.1 Strain-level prediction accuracy

**Figure 2.**
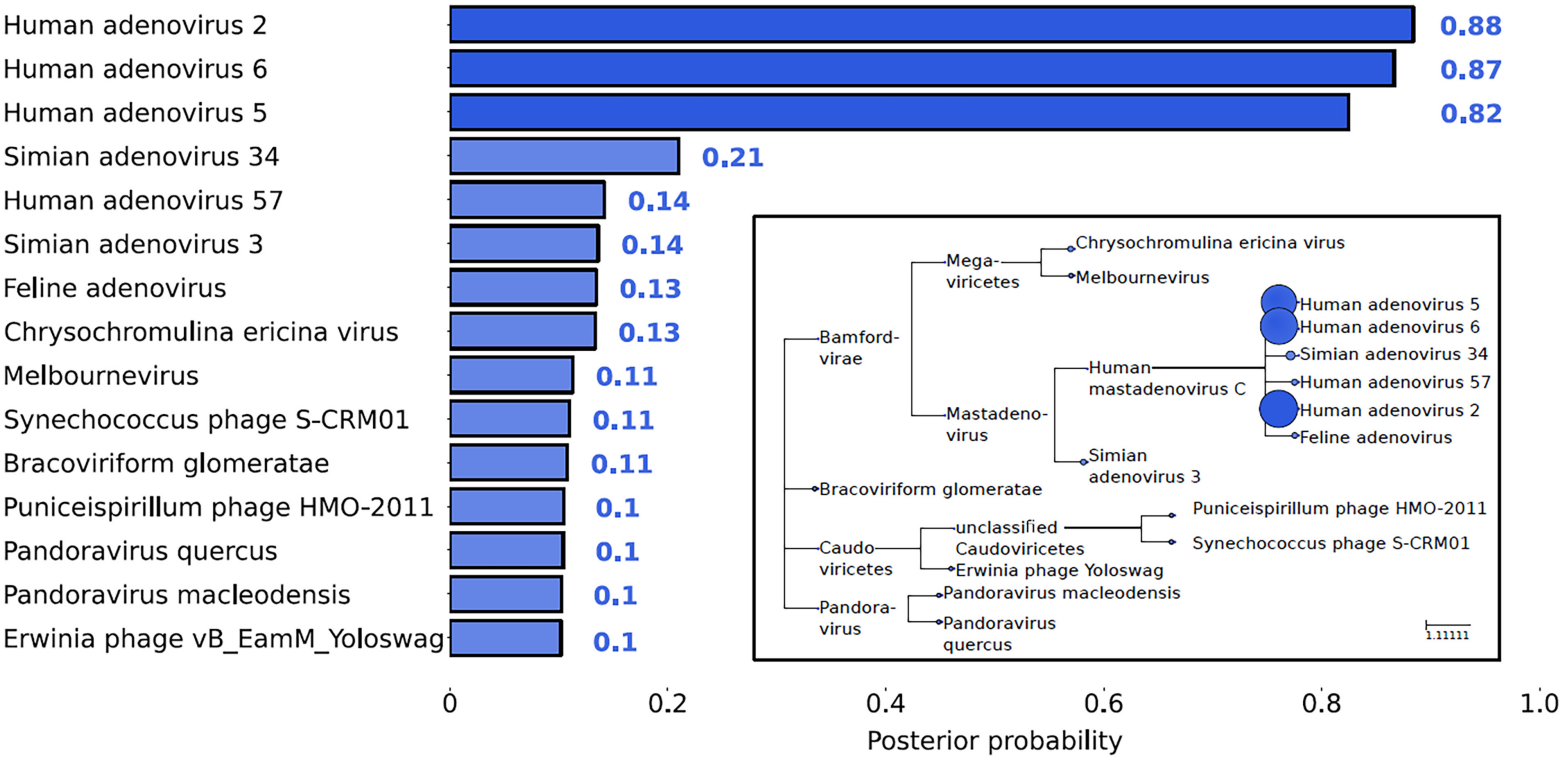
Posterior scores for the first 15 candidate taxa of the adenovirus sample (PXD004095). Human adenovirus types 2, 6, and 5 are predicted to be the most probable sources of the sample, with respective scores of 0.88, 0.87, and 0.82. An inset visualizes these findings within a taxonomic tree, effectively illustrating the relationships among the detected taxa. The circle size corresponds to the posterior level of the prediction, with larger circles representing a higher confidence.

To demonstrate that PepGM consistently predicts the correct strain in viral samples, we run the complete PepGM workflow without removing host proteins on all described samples (see Section 2.2). For the adenovirus sample, the posterior scores of the 15 highest-scoring taxa are presented in Fig. 2. A comprehensive presentation of the PepGM results for all additional samples is available in Supplementary Material S2. Figure 2 demonstrates that adenovirus strain 2 was accurately inferred from the sample, as it exhibited the highest posterior probability. Two other human mastadenovirus C strains, adenovirus 5 and adenovirus 6, also received high posterior probabilities. As depicted in the inset phylogenetic tree representation (see Fig. 2 inset), these strains lie in close taxonomic proximity to the actual strain. This might be due to their high-sequence similarity. To investigate this further, we computed the peptidome similarity of the 15 top-scoring viral strains that were co-predicted for the same sample. We used a symmetric similarity measure (Alves et al. 2018). Here, the peptidome similarity was restricted to peptides that were actually identified by the search engine, thus those peptides that are present in the graphical model, in order to more accurately represent the similarity of the detected peptidomes. We call this measure the detected peptidome similarity.

**Figure 3.**
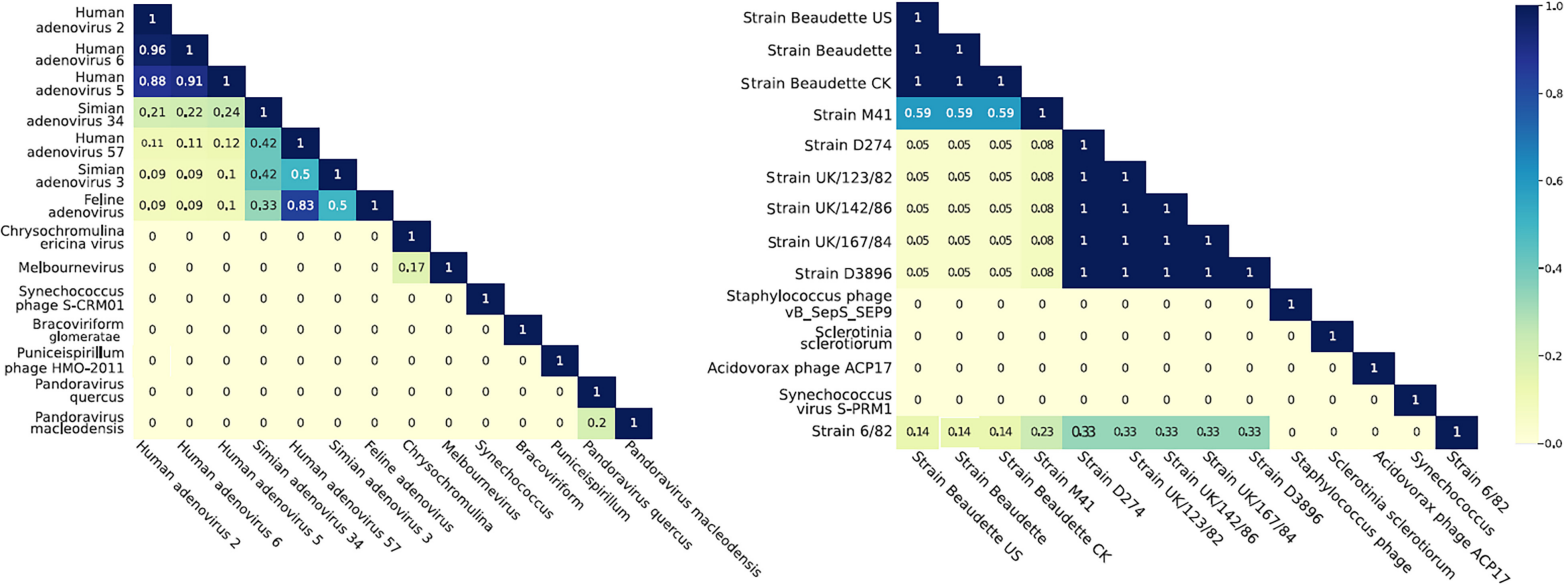
Peptidome similarity of the top-scoring viral strains for the adenovirus and the avian bronchitis sample. Peptides included for the peptidome similarity computation are restricted to the peptides that were identified by SearchGUI/PeptideShaker and included in the graphical model.

The detected peptidome similarity is calculated as follows: For a peptide set X and a second peptide set Y, $Sim_{X,Y}=\frac{X\cap Y}{\max(|X|,|Y|)}$. As expected, the three top-scoring strains, i.e., human adenovirus 2, 5, and 6, had a high detected peptidome similarity between 0.88 and 0.96 (see Fig. 3). The two other slightly higher scoring taxa (simian adenovirus 34 and human adenovirus 57 with respective posterior probabilities of 0.21 and 0.14) also had a slightly higher detected peptidome similarity (0.21 and 0.11, respectively) than other lower-scoring taxa. All other taxa included in Fig. 2 are not part of the mastadenovirus genus and do not share peptides with this genus. Their presence in the results likely stems from either wrongly identified peptides or the presence of host proteins and other impurities. The low posterior probabilities (all below 0.13) reflect this observation. A summary of the prediction results for all samples that provide details on the true viral strain, the predicted viral strain, and the computed posterior score for all analyzed samples is presented in Table 1.

**Table 1.** Summary of the taxonomic inference results for eight selected samples, utilizing the PepGM method.^a^

Sample	Strain		Posterior
PXD003013	Cowpox virus (Brighton Red)	✓	0.96
PXD014913	Cowpox virus (Brighton Red)	✓	0.88
PXD018594	SARS-CoV-2	✓	0.99
PXD025130	SARS-CoV-2	✓	0.99
PXD005104	Human herpesvirus 1 (strain F)	±	0.81
PXD002936	Avian bronchitis (Beaudette CK)	±	0.78
PXD001165	Hendra virus 1994	✓	0.99
PXD00409	Human adenovirus (strain 2)	✓	0.88

a For all samples, PepGM predicted the correct species.

✓ indicates a correct identification, ± indicates ambiguous results that include the correct strain. Posterior stands for posterior probability.

**Figure 4.**
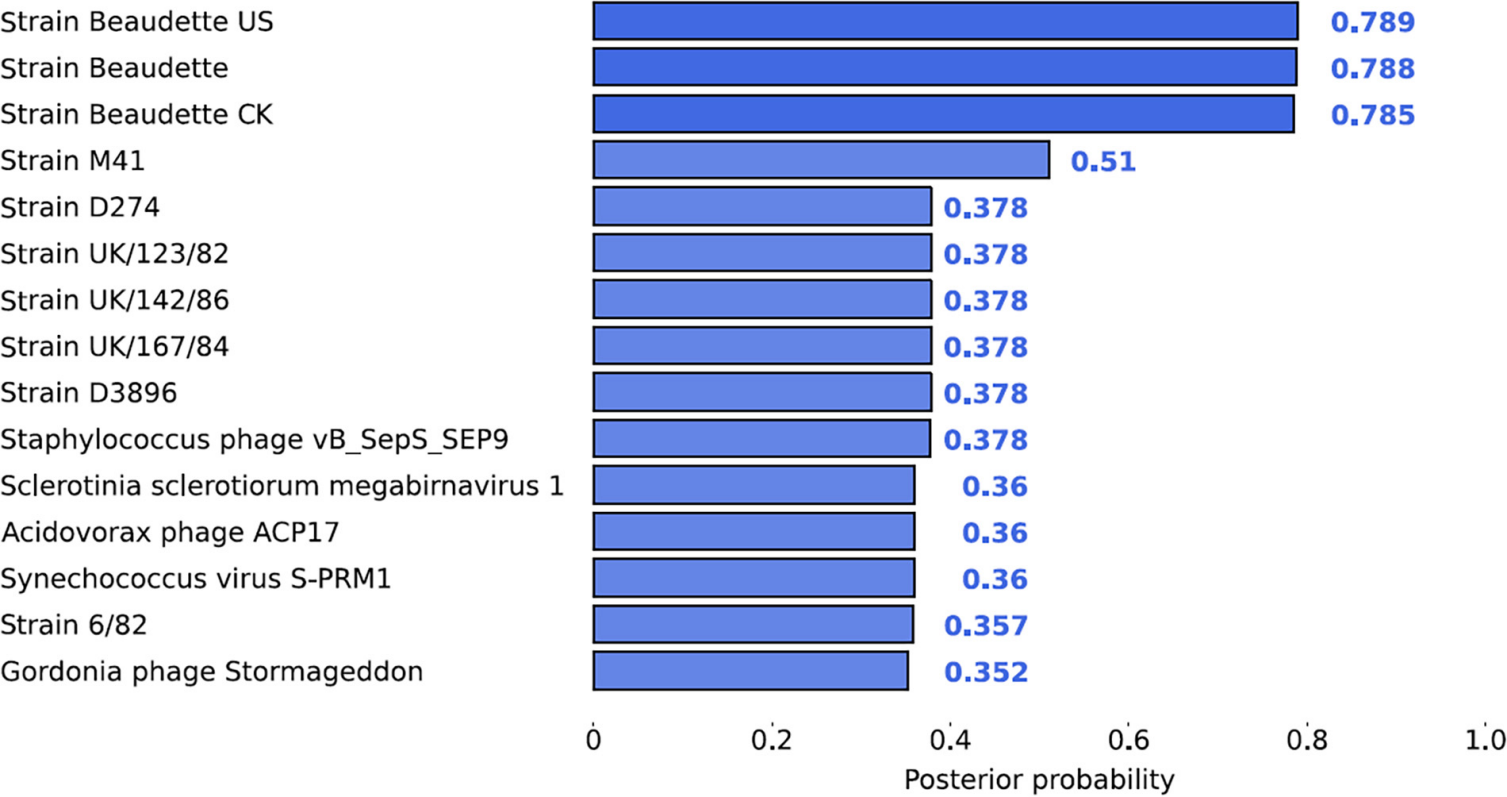
Posterior scores for the first 15 candidate taxa of the avian bronchitis sample (PXD002936). Strain beaudette subtypes US, regular (no extension) and CK are predicted to be the most probable sources of the sample, with respective scores of 0.789, 0.788, and 0.785.

PepGM predicted the correct species for all, and the correct strain for six out of eight samples, exhibiting high posterior probabilities (between 0.99 and 0.88) to true taxa. For the herpesvirus and the avian bronchitis sample, we interpret the inference as ambiguous because PepGM was neither able to resolve between closely related strains nor predict a neighboring strain (from the correct species) as present. We next investigated the potential reasons for such ambiguous identifications. Figure 4 shows the top-scoring taxa for the avian bronchitis virus (one of the ambiguously predicted strains, see Table 1 for details). Figure 4 shows that PepGM was not able to differentiate between the three avian bronchitis Beaudette subtypes: CK, US, and regular (no extension). The US and the CK strain are variants of the regular Beaudette strain that have adapted for optimal growth in different cell lines (Casais et al. 2001). Therefore, US and regular strains are taxonomically (and peptide sequence-wise) closely related. The posterior probability of each false Beaudette subtype strain (0.78–0.79) was lower than the probability that was predicted for the correct identifications in other samples (which were all above 0.88), demonstrating the accuracy of the confidence estimate based on the posterior probabilities. None of the other taxonomic inference software tools, including TaxIt, was able to identify the correct Beaudette strain; therefore, PepGM had the added benefit of reporting the lower confidence of its identification. To further explore our results, we computed the detected peptidome similarity for the avian bronchitis sample. The results depicted in Fig. 3 (right panel) show that the three avian bronchitis Beaudette strains have a detected peptidome similarity of 1, which means that all identified peptides map to all three strains. This shows that, based on the available information, no algorithm would be able to differentiate between them.

**Figure 5.**
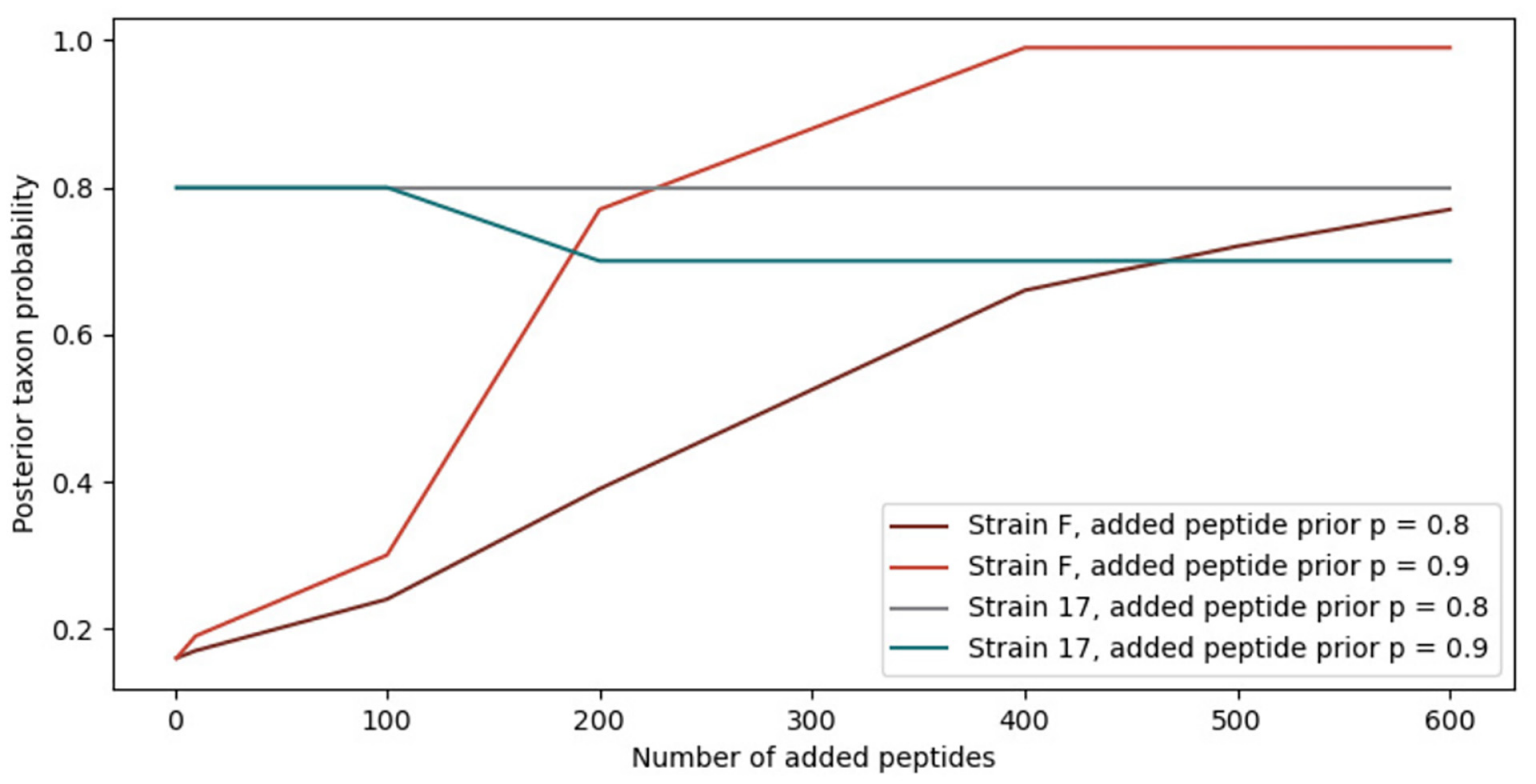
Effect of sequentially adding peptides (to the herpesvirus strain F) on the posterior probabilities of the previously highest-scoring herpesvirus strain (strain 17, gray/blue lines) and the herpesvirus strain present in the sample (strain F, red/burgundy). For the blue lines, peptides with a prior probability of *p* = 0.9 were added, while for the gray and black lines, peptides with a prior probability of *p* = 0.8 were added.

For the herpesvirus sample, PepGM predicted human herpesvirus strain 17 (refer to Supplementary MaterialFig. S7 for detailed results), while the correct strain was actually strain F. The second and third highest-scoring taxa were the neighboring herpesvirus strains RH2 and KOS. However, the true herpesvirus strain F was ranked 4th with a relatively low score of 0.15. TaxIt likewise fell short in predicting the correct strain, leading the authors to contemplate the potential for a mistaken sample annotation. However, given the clear description of the acquisition and annotation processes in the related publication (Snijder et al. 2017), this scenario appears unlikely to us. The predicted human herpesvirus strain 17, which is the most widely used reference strain for human herpesviruses, was significantly overrepresented in the general NCBI reference database with 2553 entries, as compared to strain F, which had only 282 entries in NCBI Protein. Again, the score PepGM attributed to its identification, about 0.81, was lower than the lowest score of the correct identifications in other samples, highlighting once more the usefulness of the attributed scores. To demonstrate the extent to which misidentification may be associated with an enlarged reference database size, we simulated the growth in size by progressively adding peptides to the correct herpesvirus strain (strain F) node within the graph. Initially, the herpesvirus strain 17 taxon node neighbors 514 peptide nodes, whereas the accurate strain, strain F, features 82 peptide nodes. We sequentially added 1, 10, 100, and up to 600 peptide nodes to the strain F node, with a prior probability of either p=0.8 or p=0.9. The impact of additional peptides on the posterior probability of strain 17 and strain F is presented in Fig. 5. For peptides with p=0.8, Fig. 5 reveals that with an increasing number of added peptides, the posterior probability for strain F (the correct strain) increased too (burgundy line), whereas the posterior probability for strain 17 remained unchanged (gray line). Thus, increasing the reference database size leads to an increase in the posterior taxon probability for the correct taxon. This effect was even more evident when adding higher confidence peptides with p=0.9 (red and blue lines). Here, the posterior probability of strain F increased from 0.15 to 0.99 if more than 400 peptides are added (red line), while the posterior probability of the previously highest-scoring strain 17 decreased slightly from initially around 0.8 to 0.7 (blue line). Therefore, not only peptide number (related to database size and detected peptides) but also the associated priors influenced posterior probabilities, as intended by the design of PepGM.

To assess the impact of missing species information on PepGM, we examined the cowpox virus (sample PXD014913), the adenovirus, and the Hendra virus strain inference results using a modified viral reference database, where entries corresponding to the correct species and strain (if available) have been removed (see Supplementary Material Fig. 15 for plots). For the cowpox virus, a range of orthopoxviruses was predicted with confidence scores around 0.6. Comparable results can be observed for human adenovirus 2, which displayed a series of low-confidence assignments of strains from the species human mastadenovirus D, while the correct species was human mastadenovirus C. When the correct species (human mastadenovirus C) was removed from the database, a series of low-confidence identifications from the same genus suggests the presence of a microorganism from that genus but the correct species is either unknown or absent in the database. In the case of the Hendra virus, however, the species Nipah henipavirus from the correct genus henipavirus was inferred with a confidence score of 0.86 (the correct strain, Hendra henipavirus, was scored a posterior of 0.99). In this scenario, the PepGM score did not provide a definitive signal that the prediction might be incorrect.

Lastly, we aim to offer an intuitive understanding of interpreting PepGM posterior probabilities for inferred taxa when multiple taxa exhibit high posterior probabilities. Take, for instance, the adenovirus sample for which three taxa had relatively high posterior probabilities. This should not be interpreted as a diminished confidence in the highest-scoring taxon. Rather, since all three high-scoring taxa possessed a high sequence similarity, they must, statistically speaking, have a high likelihood of being the true taxon, and thus receive high scores. Nevertheless, one can have equal confidence in identifications with only one high-scoring taxon as in identifications with multiple high-scoring candidates, as equal posterior probabilities indicate an equivalent likelihood of presence.

### 3.2 Effect of unique or shared peptides and their score

To explore the impact of unique or shared peptides on the prediction, we run PepGM using the graphical model derived from the avian bronchitis sample with various manually added peptides. The avian bronchitis graph structure is ideally suited for this analysis because all three potential strains have identical detected peptidomes (see Fig. 3, right panel), rendering them indistinguishable within the graph structure. These peptide constellations are:

1. A single high-confidence peptide node (score 0.9) was added to the Beaudette US strain.
2. A single medium-confidence peptide node (score 0.7) was added to the Beaudette US strain.
3. A single lower-confidence peptide node (score 0.5) was added to the Beaudette US strain.
4. A shared medium-confidence peptide node (score of 0.7) was added to the Beaudette CK and US strains.

Table 2 shows how the additional peptides affect the posterior probabilities. By adding a single high-confidence peptide to the avian bronchitis Beaudette US strain, its score increased to 0.92, while the scores for the two neighboring strains decreased to 0.62. This demonstrates that a single high-confidence peptide is sufficient for PepGM to identify the correct strain without entirely dismissing the other two potential strains. Adding a medium-confidence peptide did not raise the score of 0.78 for the Beaudette US strain but it reduced the scores attributed to the other strains to 0.58. This indicates that PepGM remains capable of identifying the correct strain even with the addition of a medium-confidence peptide. Adding a peptide with an equal likelihood of being correct or incorrect did not improve the prediction. Additionally, the introduction of a medium-confidence peptide shared by two neighboring strains increased their respective score to 0.8, while decreasing the score assigned to the third neighboring strain. These findings highlight that PepGM meticulously evaluates peptide scores and that, based on their confidence, a single unique peptide can effectively differentiate strains with identical detected peptidomes.

**Table 2.** Change in posterior probabilities for the three best scoring strains for the avian bronchitis sample when manually adding peptides with differing scores.

Peptide constellation	1	2	3	4
Beaudette US	0.92	0.78	0.78	0.8
Beaudette CK	0.62	0.58	0.78	0.8
Beaudette	0.62	0.58	0.78	0.61

We also investigated, for the herpesvirus, the avian bronchitis, and the Hendra virus, the effect of setting different FDR thresholds: 1%, 5%, and 20%. These effects were very limited, as shown in the Supplementary Fig. S16. As described for the manually added peptides, the taxonomic inference and scores computed by PepGM were dominated by high-confidence peptides, which explains the insensitivity to changes in the FDR threshold.

### 3.3 Effect of optional host filtering

PepGM provides the option to filter for host proteins before the database search. In some cases, such as for both SARS-CoV-2 samples (see Supplementary Materials S2, Figs. S9–S12), the optional filtering step does not or only slightly alter the prediction. For the filtered human adenovirus sample, PepGM was not able to differentiate between the human adenovirus strains 2, 6, and 5 anymore (see Supplementary Material S2Fig. S1). It is plausible that filtering for potential host and contaminant peptides eliminated some peptides that were also associated with one of the strains in question. A similar effect can be seen in the prediction results of the avian bronchitis sample (see Supplementary MaterialFig. S2). For purified viral samples, as seen in all the samples analyzed in our benchmark, we recommend opting against the host filtering step.

### 3.4 Grid search of graphical model parameter

We also investigated the parameters α,β, and γ that are found to maximize the empirical metric (see Section 2). For each sample, the set of best-fitting parameters is shown in Table 3. For the parameter γ, representing the prior probability for a taxon to be present, the grid search was conducted in the range γ∈{0.1,0.3,0.5}. Here, setting γ=0.5, which corresponds to a uniform distribution, encodes the knowledge that no prior information is available on any taxon. Table 3, however, shows that for most samples γ=0.1 is the selected parameter. This observation stems from the fact that we are dealing with single-organism samples in which, indeed, the true prior for most taxa would be close to 0, as they are not present. For avian bronchitis, the grid search identified a maximizing prior of γ=0.3. In this case, the value is influenced by the simultaneous “presence” of the three Beaudette avian bronchitis strains, among which all peptides are shared.

**Table 3.** Grid search results for the noisy OR conditional probability tables.

Sample	Strain	α	β	γ
PXD025131	SARS-CoV-2	0.4	0.7	0.1
PXD005104	Human herpesvirus 1 strain F	0.01	0.4	0.1
PXD002936	Avian bronchitis (Beaudette CK)	0.05	0.1	0.3
PXD001165	Hendra virus 1994	0.05	0.05	0.1
PXD004095	Human adenovirus strain 2	0.01	0.1	0.1

The best-fitting parameter differs for each sample and is most likely determined by multiple factors across the experimental workflows, e.g., the variability in detection accuracy and the inherent differences in the peptide-taxon mappings between different species or genera. The parameter tends to be more similar for different samples from the same strain (for which different experimental workflows were used), such as the two cowpox viruses or the two Sars-CoV-2 samples. Therefore, for strains from a given species or strain, the user is suggested to use the same parameter set to ensure the comparability of taxonomic scores. For the parameters α (corresponding to the ‘emission probability’ of a peptide given its parent taxons presence) and β (the false detection probability of a peptide), the observed ranges, namely α∈[0.01,0.4], β∈[0.01,0.7], and α≤β, are less intuitive and harder to explain. If interpreted according to their meaning in the noisy OR model, the parameters suggest that the probability of observing a peptide, given the presence of its parent taxon, is very low and that many peptides are detected erroneously. However, we modeled a complex physical process with a simple noisy OR approach and made the assumption that α and β are the same for all peptides, both of which are strong simplifications. It is, therefore, more likely that our simplified noisy OR model is not an accurate representation of the underlying peptide detection, which impairs the direct interpretation of the α and β values. Nevertheless, given the effectiveness of PepGMs taxonomic inference, the use of this simplified model remains justified.

### 3.5 Comparison to the identification results of TaxIt

We have already mentioned other pipelines that are able to infer taxa on strain level. One of these, TaxIt, is the only tool that is able to provide strain-level resolution for a broad range of samples (Kuhring et al. 2020). We, therefore, compared the prediction performance, run time, and memory use of PepGM and TaxIt. Table 4 shows that PepGM predicted the viral strain equally accurately as TaxIt for six out of eight viral samples. In the two cases where the predictions of PepGM were ambiguous (the avian bronchitis sample) or correct regarding the species but incorrect regarding the strain (the herpesvirus sample), the predictions of TaxIt were incorrect or ambiguous, too. The challenges faced by TaxIt in accurately inferring the taxonomy of these two samples likely stem from the same factors affecting PepGM: in the case of avian bronchitis, the detected peptidomes were too similar, while for the herpesvirus, the incorrect strain was overrepresented in the reference databases.

**Table 4.** Inference accuracy of PepGM and TaxIt for all benchmark samples comparing run time and memory.

Sample	Strain		PepGM	TaxIt
PXD003013	Cowpox virus (Brighton Red)	Taxonomy	✓	✓
		Run time	26:35:25	0:19:51
		Memory	3.6 GB	4 GB
PXD014913	Cowpox virus (Brighton Red)	Taxonomy	✓	✓
		Run time	04:40:39	0:39:07
		Memory	4.2 GB	2.4 GB
PXD018594	SARS-CoV-2	Taxonomy	✓	X
		Run time	0:49:09	–
		Memory	3.9 GB	–
PXD025130	SARS-CoV-2	Taxonomy	✓	X
		Run time	0:38:17	–
		Memory	2.9 GB	–
PXD005104	Human herpesvirus 1 (strain F)	Taxonomy	±	±
		Run time	08:32:32	0:37:11
		Memory	3.4 GB	3.8 GB
PXD002936	Avian bronchitis (Beaudette CK)	Taxonomy	±	±
		Run time	0:34:05	01:30:23
		Memory	3.7 GB	2.7 GB
PXD001165	Hendra virus 1994	Taxonomy	✓	✓
		Run time	01:48:08	01:35:12
		Memory	3.9 GB	2.8 GB
PXD004095	Human	Taxonomy	✓	✓
	adenovirus (strain 2)	Run time	02:20:04	03:06:57
		Memory	5 GB	7.7 GB

✓ means correct identification, ± means correct species but incorrect strain or ambiguous strain inference, and X means that the inference failed.

Notably, PepGM is able to achieve a similar taxonomic resolution using only the peptide search results from a single database search, while TaxIt relies on a second, refined search. PepGM has the added benefit of providing confidence scores, indicating when its inference might be less confident. For the two coronavirus samples, TaxIt did not output results—this is due to its use of the NCBI Entrez API and the currently not updated NCBI taxonomy structure for certain taxa in the NCBI nodes dump files. Future updates from NCBI could potentially resolve this issue. TaxIt predicts the SARS-CoV species and subsequently attempts to download all corresponding strain-level proteins via the API. However, the 260 associated strains were too large to be queried through the API. In contrast, PepGM, which utilizes only the NCBI protaccession2TaxID file to infer species candidates, directly recognizes SARS-CoV-2 as a candidate species. As a result, PepGM queries significantly less information through the API, enabling it to complete the analysis faster. The total run time and memory usage of PepGM and TaxIt are shown in Table 4. The memory usage of PepGM and TaxIt is comparable. As for run time, a single conclusion cannot be drawn for all samples. In our benchmark, TaxIt’s run time was often dominated by the time required to download individual protein entries through the NCBI API. In contrast, PepGM queries whole proteomes per organism at once, making its run time more dependent on the number of strains and peptides included in the graph. Building the factor graph needs querying pairwise peptide–taxon proteome comparisons, and the more complex statistical computations, where messages are updated iteratively for each graph edge, result in slower performance as the graph size increases. PepGM’s grid search can, in theory, be executed completely in parallel and could thus be accelerated markedly by using more CPUs. If reducing the number of points searched through in the grid search is not feasible, one could choose to limit the search points, potentially affecting result accuracy but leading to a significant speed up. For two samples, cowpox virus PXD003013 and the cowpox virus sample, the run time was a lot longer. For both, it was dominated by few belief propagation runs on certain parameter sets that took a very long time to converge. In fact, some initial variable sets can cause oscillations in the messages passed through belief propagation, slowing down and possibly impeding convergence (Knoll et al. 2015). Adding some kind of optional damping (Pretti 2005) could, in the future, speed up the belief propagation algorithm for the parameter sets in question.

## 4 Comparison to peptide similarity scoring scheme

To further evaluate the usability of PepGM, we compared the results of a very simple peptide similarity scoring to PepGM’s results. We used the identified peptidome of a strain (as described previously) and compared it against the expected peptidome of the correct strain. The peptidome similarity of a detected peptide set X and theoretical peptide set Y is then given $Sim_{X,Y}=\frac{X\cap Y}{\max(|X|,|Y|)}$. Table 5 compares the peptidome similarity for the two highest-scoring strains to the correct strain.

**Table 5.** Comparison of the PepGM score and the peptidome similarity score for the correct viral strain and the detected peptides mapping to the two highest-scoring strains in PepGM.

Sample	Correct viral strain	Detected viral strain	PepGM	Similarity
PXD003013	Cowpox virus (Brighton Red)	Cowpox virus (Brighton Red)	0.96	0.0058
		Orhtopoxvirus Abatino	0.21	0.0043
PXD014913	Cowpox virus	Cowpox virus (Brighton Red)	0.88	0.0065
	(Brighton Red)	ACT virus 1	0.53	0
PXD018594	SARS-CoV-2	SARS-CoV-2	0.99	0.046
		SARS Coronavirus Tor 2	0.96	0.0093
PXD025130	SARS-CoV-2	SARS-CoV-2	0.99	0.046
		SARS Coronavirus Tor 2	0.96	0.0093
PXD005104	Human herpesvirus 1 (strain F)	Human herpesvirus 1 (strain 17)	0.81	0.038
		Human herpesvirus 1 (strain RH2)	0.79	0.049
PXD002936	Avian bronchitis (Beaudette CK)	Avian bronchitis (Beaudette CK)	0.78	0.029
		Avian bronchitis (Beaudette)	0.78	0.029
PXD001165	Hendra virus 1994	Hendra virus 1994	1	0.017
		Nipah Henipavirus	0.97	0.0095
PXD004095	Human adenovirus (strain 2)	Human adenovirus 2	0.88	0.0083
		Human Adenovirus 6	0.86	0.011

Overall, the similarities were very low because the detected peptides only cover a small fraction of the peptides that are derivable from the available reference proteomes. This is expected because only a fraction of all peptides are detected in the mass spectrometer, whereas the reference strain proteomes can be large and include uncurated proteomes (as the aim of PepGM is to make use of all strain-level information available). For most samples, the correct strain had a slightly higher peptidome similarity (between 0.1% and 3%); however, this is not true for the adenovirus sample that PepGM correctly inferred. In cases where neither PepGM nor the similarity score were able to tell both strains apart, such as for the avian bronchitis samples, PepGM predicted a lower posterior probability for the detected strains. This also applies to the human herpesvirus sample, where relying solely on peptidome similarity could lead to misleading conclusions. Moreover, the PepGM score is designed to be directly interpretable as a probability for the detected peptidome originating from a specific viral strain, and this probability score is comparable across samples, with lower values for ambiguous predictions, such as those for the avian bronchitis sample. This is not the case for the similarity score, where the ambiguous avian bronchitis strain exhibited a lower similarity.

## 5 Discussion and outlook

We have shown that PepGM consistently and reliably determines the taxonomy of viral samples with strain-level accuracy. Using a graphical model approach, PepGM makes use of peptide scores returned by database search engines or post-processing tools to calculate meaningful confidence scores for taxonomic inferences. This is especially useful in clinical settings when knowing the reliability of strain prediction impacts therapeutic decision-making.

For two of the benchmark samples, the taxonomic inferences were either ambiguous or only the correct species, but only a closely related viral strain was predicted. For avian bronchitis, PepGM could not differentiate between the three Beaudette subtypes. We showed that this was due to their detected peptidomes being identical. Since PepGM indicates lower confidence by low posterior probabilities, this remains a defensible result, especially as TaxIt was not able to resolve the ambiguity either. For the herpesvirus, PepGM identified strain 17 instead of strain F as present in the sample. No other algorithm could provide a correct taxonomic assignment for this sample (Kuhring et al. 2020). As previously discussed, the false prediction can be explained by the over-representation of strain 17 in the available proteome references. Possible solutions to explore might include the normalization of peptide or taxon priors depending on proteome size, the number of proteins in the database, or the introduction of weighted edges in the graphical model.

In general, PepGM does not eliminate the reliance on public reference databases. By integrating the results of a search against the curated viral reference with the query of all available strain proteomes, PepGM aims to make the best use of the available information. Uncurated references, which are included by querying all available strain-level reference proteomes, are prone to errors (Tao et al. 2020). Our graphical model approach, which accounts for the probability of false peptide identifications (and taxa inference), can partially mitigate this issue. A possible extension for PepGM is the inclusion of protein-level information: the confidence in the presence of a parent taxon might be higher if its identified peptides spread across multiple proteins than if they come from one single protein. One could also imagine providing an entire protein-centric version of PepGM. However, an issue stemming from the uncurated (but more strain-resolved) viral proteome databases arises: the proteome references available are highly redundant. For example, Sars-CoV-2 currently has over 29 million uncurated protein sequences (each with its own accession number) when the virus itself consists of 29 proteins only. Smart prefiltering is necessary to avoid adding noise instead of additional information to the graphical model. As long as public proteome databases keep on focusing on model organisms, a peptide-centric approach is not feasible. When the correct species was removed from the reference database, PepGM predicted various species from the correct genus with low-confidence scores, except for the Hendra virus, where the Nipah henipavirus was incorrectly inferred—still with a slightly lower probability. This demonstrates that the score provided by PepGM accurately reflects the accuracy of a taxonomic assignment. However, it also indicates that setting a threshold score above which PepGM is certain of its assignment would be ineffective. In general, multiple taxonomic assignments with similar low scores may suggest that the correct strain or species is not present in the database used. On the other hand, a few high but closely scored taxonomic assignments could indicate an inability to resolve between neighboring strains, as observed in the avian bronchitis sample.

Upon closer inspection of the graphical model parameters obtained from the grid search, it becomes evident that the values of parameters α and β do not hold any meaning in the noisy OR model. Future development could focus on utilizing peptide-specific α and β parameters, potentially based on factors such as the prediction of peptide probability after tryptic cleavage (Fannes et al. 2013) for α, or posterior error probabilities derived from PSMs but aggregated at the peptide level for β.

Compared to other available tools that can be used for strain-level inference of viral proteome samples, PepGM reaches at least the taxonomic resolution of TaxIt, the previously best-performing approach, with the additional benefit of providing confidence scores. Memory-wise, PepGM and TaxIt are comparable, but for the run time, there are sample-specific differences. The run time of PepGM, except for two samples, did not exceed 4 hours. This can still be a hindrance in clinical settings, where fast decisions need to be made. A speed-up can be achieved by improving the belief propagation algorithm. As mentioned previously, certain initial variable sets cause oscillations to slow down while impeding convergence. Damping these messages might be a future solution, as was done for the protein inference algorithm based on belief propagation (Pfeuffer et al. 2020).

Currently, PepGM is restricted to the taxonomic inference of viral samples; however, the graphical model approach can be extended to encompass all types of organisms. In the clinical context, strain-level inference of pathogenic bacterial samples is particularly relevant. To facilitate this, PepGM could be expanded by incorporating a general bacterial reference database, such as RefSeq for bacteria, and including peptide–taxon mapping for bacteria as well. Due to the necessity of downloading strain-level peptides through the NCBI API and the fact that PepGM downloads strain-level information for all target species included in the graph, it would likely be essential to establish local database options to optimize speed and bypass the NCBI API. Another alternative to enhance PepGM is to integrate it with pre-digested peptide databases like Unipept, where tryptic peptides are already taxonomically annotated. Additionally, metaproteomics presents another potential area of application, where complex microbial mixtures require taxonomic and functional identification. In this context, peptide–taxon and peptide–function relationships can be represented in bipartite graphs, allowing for the application of a similar graphical model. However, the metric for determining the best graphical model parameters would need to be adapted for the simultaneous presence of multiple organisms.

Lastly, the application of PepGM for strain-level identification of viral proteomes encounters a practical challenge due to the small size of viruses and the consequently low abundance of viral proteins.

## 6 Conclusion

In this work, we have presented PepGM, a new graphical model-based workflow for the strain-level taxonomic assignment of viral proteome samples. We have shown that PepGM is able to consistently provide accurate taxonomic inference with associated, meaningful confidence scores, a feature that could be especially helpful in the clinical context when medical decisions have to be made based on the confidence of the identification results. More generally, we have demonstrated the usability of graphical models for the taxonomic inference of proteomes samples using the restricted example of viral samples.

## Supplementary Material

btad289_Supplementary_Data
