# Supplementary material for: PepGM: a probabilistic graphical model for taxonomic inference of viral proteome samples with associated confidence scores
Source: Bioinformatics. 2023 May 2;39(5):btad289. doi: 10.1093/bioinformatics/btad289 (PMC10182852; doi:10.1093/bioinformatics/btad289)
Supplement: btad289_Supplementary_Data [file btad289_supplementary_data.pdf]

Supplementary material for PepGM: A probabilistic graphical model for taxonomic inference of viral proteome samples with associated confidence scores

Tanja Holstein,[†,‡,¶] Franziska Kistner,[†] Lennart Martens,[‡,¶] and Thilo Muth[*,†]

†*s.3 eScience, Bundesanstalt für Materialforschung und - Prüfung, Berlin, Germany*

‡*VIB-UGent center for Medical Biotechnology, VIB, Belgium*

¶*Department of Biomolecular Medicine, Ghent University, Belgium*

E-mail: thilo.muth@bam.de

Contents

S1 Libraries used for PepGM **3**

S2 Graphical model architecture details **3**

S3 Search parameters for the publicly available samples **5**

Human adenovirus 2 sample 5

Avian bronchitis (Beaudette CK) sample 5

Human herpesvirus 1 strain F 6

Hendra virus sample 6

Cowpox virus strain Brighton Red 7

SARS-CoV-2 7

S4 Additional tables and figures 8

Table 1: Packages used in the PepGM workflow

Package name	reference
numba	[1]
Ete3	[2]
mmh3	[3]
pandas	[4]
numpy	[5]
networkx	[6]
scipy	[7]
Biopython	[8]
matplotlib	[9]
seaborn	[10]

S1 Libraries used for PepGM

S2 Graphical model architecture details

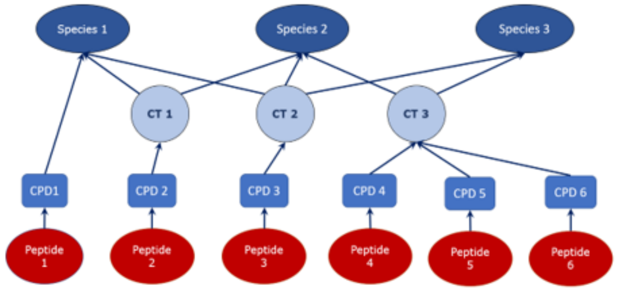

Figure 1: Example of a PepGM graphical model with three taxa (species) and 6 peptides.

Figure 1 shows an example of the graphical model architecture used by PepGM. Here, peptide 1 maps to species 1, peptide 2 to species 1 and 2, peptide3 to species 1, 2 and 3, and peptides 4, 5 and 6 to species 2 and 3.

Each peptide node gets attributed its own conditional probability table (CPD). The values in the CPD depends on the noisy-OR model parameters described in the main manuscript in Equation 1. The taxon variables n and peptide variables P are binary, as peptides can be either present or absent. Below, we give an example of an initialized conditional probability table for the parameters $\alpha = 0.8$ and $\beta = 0.3$, for peptide 2 in Figure 1. The probabilities

Table 2: Example of the conditional probability distribution table for peptide 2, $p(P) = p(P|N = n)$

	$n = 0$	$n = 1$	$n = 2$
$P = 0$	0.3	0.046	0.009
$P = 1$	0.1	0.29	0.324

in the table were calculated according to equation 1 from the main manuscript and subsequently normalized.

Further initial values are the prior probabilities for the taxon variables, were the probability for a taxon to be present is $p(N = 1) = \gamma$ and the probability for a taxon to be absent is $p(N = 0) = 1 - \gamma$. For a peptide variable, it's initial probability to be present corresponds to the score it was attributed by the database search engine.

Figure 1 additionally depicts the convolution tree nodes. These connect multiple taxon variables should they share the same peptides. The convolution tree nodes allow to efficiently execute the belief propagation algorithm and represent additive factors.[11]

Once the graphical model is initialized according to Equation 1 in the main manuscript and Figure 1, we perform belief propagation. Belief propagation performs approximate Bayesian inference. The algorithm operates by passing messages between nodes in the graph, in this case the peptides, CPD, CT and taxon nodes, until convergence is reached. The messages represent the current estimate of the marginal probability distribution over a variable, and they are updated iteratively based on the information available from the neighboring nodes. For example the message sent from the CPD in table 2 to the convolution tree node CT1 represents the conditional probability tables current estimate of the probability for there to be either 0, 1 or 2 parent taxa to peptide 2. The belief propagation algorithm was first described for exact inference on tree structure networks by Pearl in 1982,[12] who subsequently demonstrated it to be a good approximation for general graphs as well.[13] Since then, this algorithm has been used extensively[14] and a comprehensive mathematical description can be found in many textbooks.[15]

To accelerate convergence, PepGM uses a version of the belief propagation algorithm called residual belief propagation,[16] where the messages who have changed the most in one iteration get sent first, as these will have the largest impact current beliefs. Since the graph may contain loops, we use the loopy version of residual belief propagation.

S3 Search parameters for the publicly available samples

Human adenovirus 2 sample

The human adenovirus 2 sample was downloaded from PRIDE (PXD004095) and the sample 20140812_LC1_Sara_24h_MH+AL_10-03.raw was selected for analysis. We converted the .raw file to an .mgf file using the ProteoWizard MSConvertGUI (version 3.0.21232). In accordance with the original processing protocol, for the SearchGUI parameters, we used a tryptic search, 2 allowed missed cleavages, a fragment ion mass tolerance of 20ppm and precursor ion mass tolerance of 4.5ppm. For the modifications, we used the fixed modification cysteine carbamidomethylation (+57 Da) as well as additional variable modifications methionine oxidation (+16 Da), acetylation of the protein N-terminus (+42 Da) and phosphorylation of serine, threonine and tyrosine (+80 Da). The host proteome - homo sapiens, was downloaded from Uniprot and had 26591 entries.

Avian bronchitis (Beaudette CK) sample

The avian bronchitis (Beaudette CK) sample was downloaded from PIRDE (PXD002936) and we used the sample BeauR2.raw for analysis. We converted the .raw file to an .mgf file using the ProteoWizard MSConvertGUI (version 3.0.21232). For the SearchGUI parameters, we used a tryptic search, 2 allowed miscleavages, a precursor ion tolerance of 100ppm, a fragment ion tolerance of 0.5Da and a charge of 2-6)in accordance with the default settings of X!Tandem). As fixed modification, we specified cysteine carbamidomethylation (+57 Da). The host proteome, chicken or gallus gallus, was downloaded from Uniprot and had 2618

entries.

Human herpesvirus 1 strain F

The human herpesvirus 1 (strain F) sample was downloaded from Pride (PXD005014) and we selected the sample OR10_20151030_EC_HHV1_A_02.raw for analysis. We converted the .raw file to an .mgf file using the ProteoWizard MSConvertGUI (version 3.0.21232). For the SearchGUI parameters, in accordance with the original processing parameters, we used a precursor ion tolerance of 100ppm, a fragment ion tolerance of 0.5Da and a charge of 2-6 (in accordance with the default settings of X!Tandem). As fixed modification, we specified cysteine carbamidomethylation (+57 Da) and variable modification methionine oxidation (+16 Da). The host proteome - homo sapiens, was downloaded from Uniprot and had 26591 entries.

Hendra virus sample

Hendra henipavirus samples of the strain horse/Australia/Hendra/1994 were downloaded from Pride (PXD001165) and we selected the sample Slice_10_human.raw for analysis. We converted the .raw file to an .mgf file using the ProteoWizard MSConvertGUI (version 3.0.21232). For the SearchGUI parameters we used a precursor ion tolerance of 100ppm, a fragment ion tolerance of 4.5ppm and a charge of 2-6 (in accordance with the default settings of X!Tandem). As fixed modification, we specified cysteine carbamidomethylation (+57 Da). As the sample is SILAC labeled we additionally specified Arginine 13C(6) 15N(4), and Lysine 13C(6) 15N(2) as variable modifications. The host proteome - homo sapiens, was downloaded from Uniprot and had 26591 entries.

Cowpox virus strain Brighton Red

We downloaded two Cowpox Virus (strain Brighton Red) samples from Pride.

The first one is the sample PXD003013, where we selected the spectrum CPXV_BR_2.RAW for analysis. We converted the .raw file to an .mgf file using the ProteoWizard MSConvertGUI (version 3.0.21232). In accordance with the original processing protocol, for the SearchGUI parameters we used a tryptic search, a precursor ion tolerance of 4.5ppm, a fragment ion tolerance of 0.5Da. As fixed modification, we specified cysteine carbamidomethylation (+57 Da) as well as additional variable modifications methionine oxidation (+16 Da), acetylation of the protein N-terminus (+42 Da). The host proteome - homo sapiens, was downloaded from Uniprot and had 26591 entries.

The second is from the Pride project PXD014913, where we selected the sample CPXV-0,1MOI-supernatant-HEp-24h.mgf for analysis. We converted the .raw file to an .mgf file using the ProteoWizard MSConvertGUI (version 3.0.21232). In accordance with the original processing protocol. For the SearchGUI parameters we used a tryptic search, a precursor ion tolerance of 4.5ppm, a fragment ion tolerance of 0.5Da. As fixed modification, we specified cysteine carbamidomethylation (+57 Da) as well as additional variable modifications methionine oxidation (+16 Da). The host proteome - homo sapiens, was downloaded from Uniprot and had 26591 entries.

SARS-CoV-2

The first SARSs-CoV-2 sample was downloaded from Pride (PXD018594) ad we selected the sample Q10467_MS20-17_CoV2_J4(MOI-01)r.mgf for analysis. According to te original processing protocol, we used a tryptic search, a precursor ion tolerance of 4.5ppm, a fragment ion tolerance of 0.5Da. As fixed modification, we specified cysteine carbamidomethylation (+57 Da) as well as additional variable modifications methionine oxidation (+16 Da).

We downloaded the second SARS-CoV-2 sample from Pride (PXD025130) and selected the sample Q26431_MS20-025_Virus-purif_1.mgf for analysis. According to the original process-

ing protocol, we used a tryptic search, a precursor ion tolerance of 4.5ppm, a fragment ion tolerance of 0.5Da. As fixed modification, we specified cysteine carbamidomethylation (+57 Da) as well as additional variable modifications methionine oxidation (+16 Da)

S4 Additional tables and figures

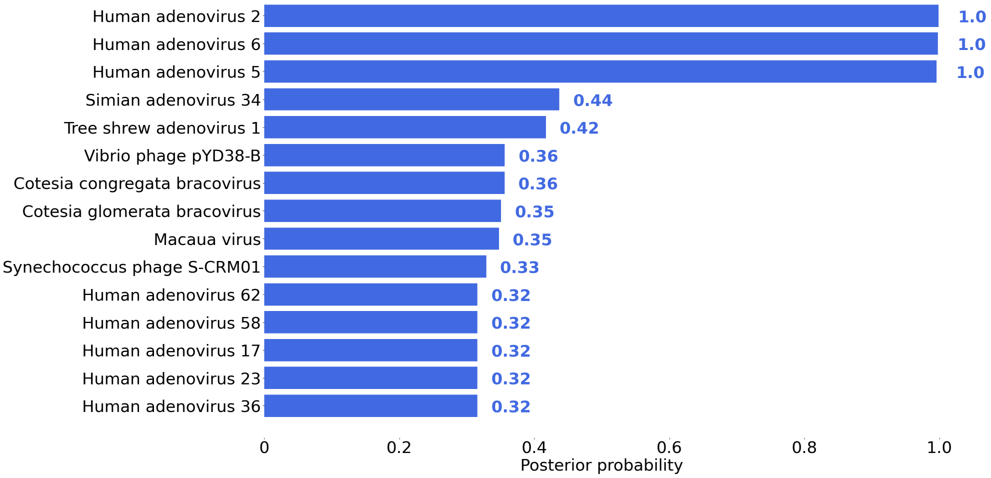

Figure 2: PepGM identification Results for the adenovirus sample with additional host filtering. Bar plot representation of the 15 highest scoring taxa, with the rounded attributed score written next to each bar.

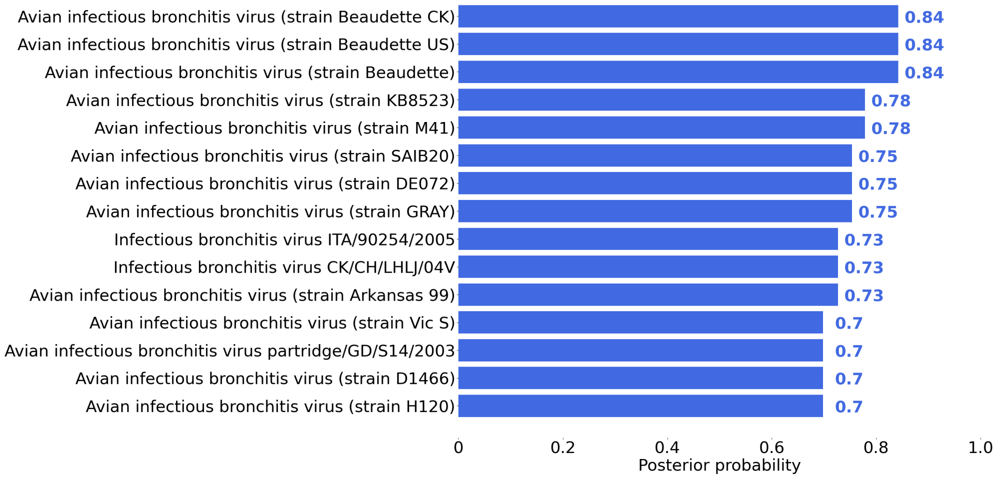

Figure 3: PepGM identification Results for the avian bronchitis sample with additional host filtering. Bar plot representation of the 15 highest scoring taxa, with the rounded attributed score written next to each bar.

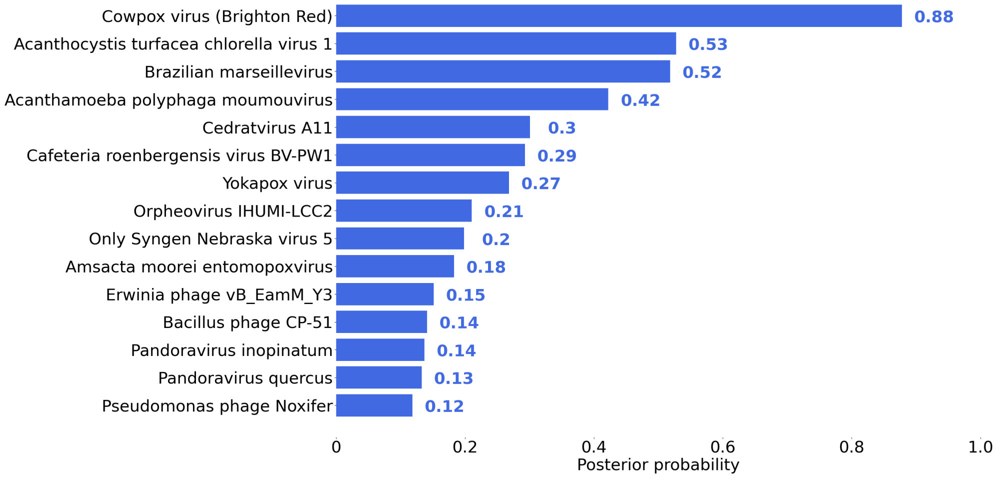

Figure 4: PepGM identification Results for the cowpox sample PXD014913 Bar plot representation of the 15 highest scoring taxa, with the rounded attributed score written next to each bar.

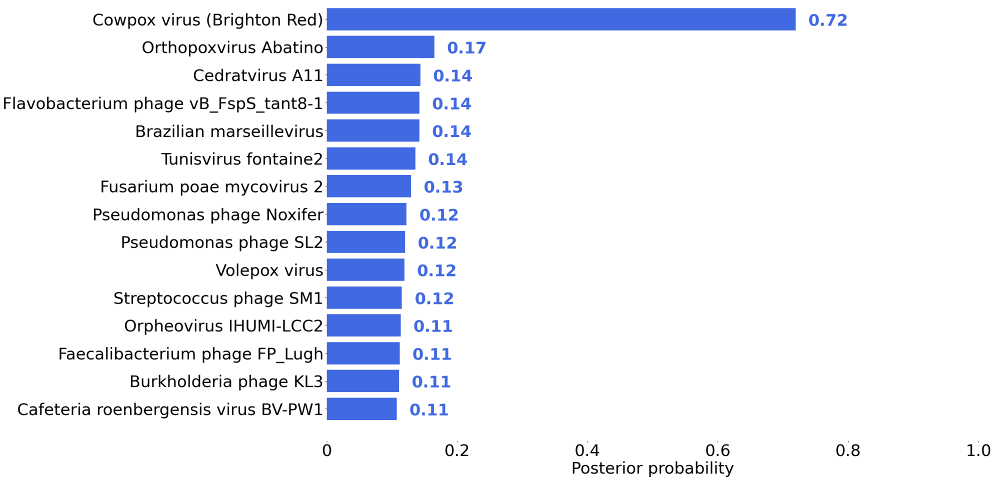

Figure 5: PepGM identification Results for the cowpox sample PXD014913 with additional host filtering. Bar plot representation of the 15 highest scoring taxa, with the rounded attributed score written next to each bar.

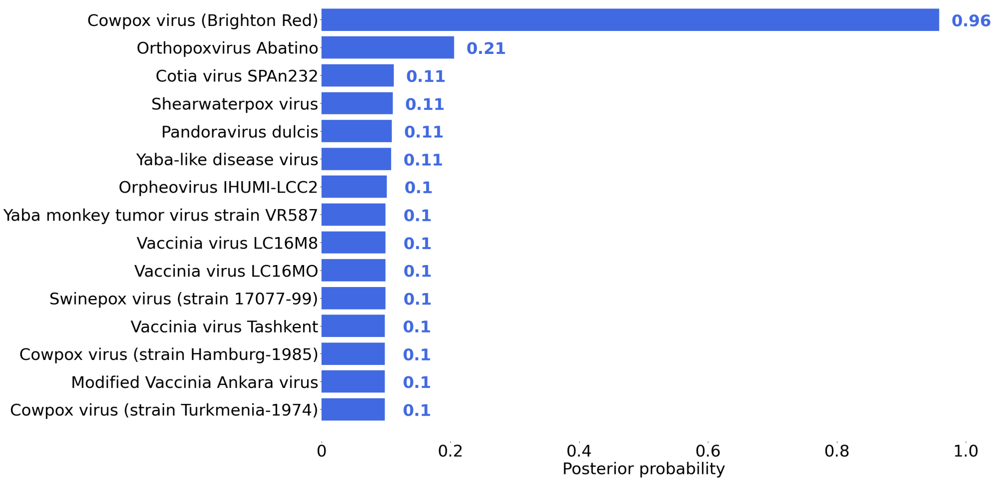

Figure 6: PepGM identification Results for the cowpox sample PXD003013 Bar plot representation of the 15 highest scoring taxa, with the rounded attributed score written next to each bar.

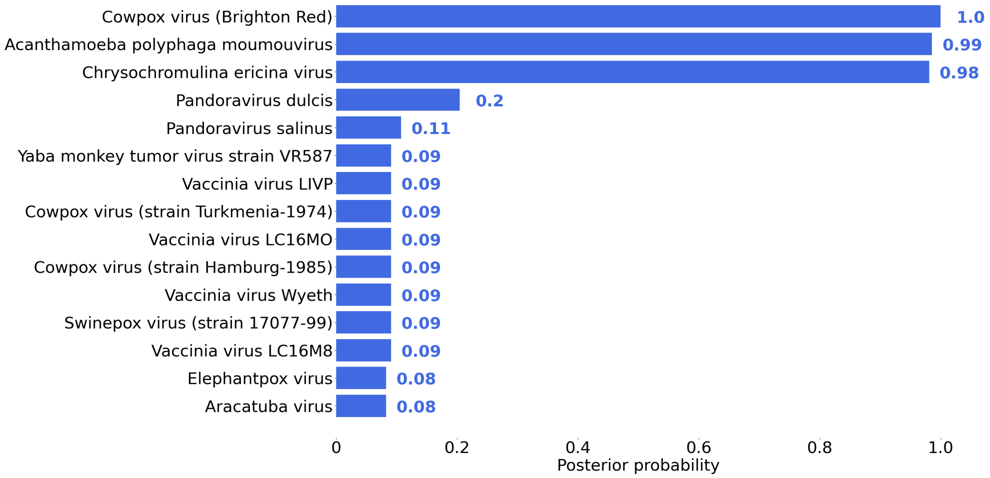

Figure 7: PepGM identification Results for the cowpox sample PXD003013 Bar plot representation of the 15 highest scoring taxa, with the rounded attributed score written next to each bar.

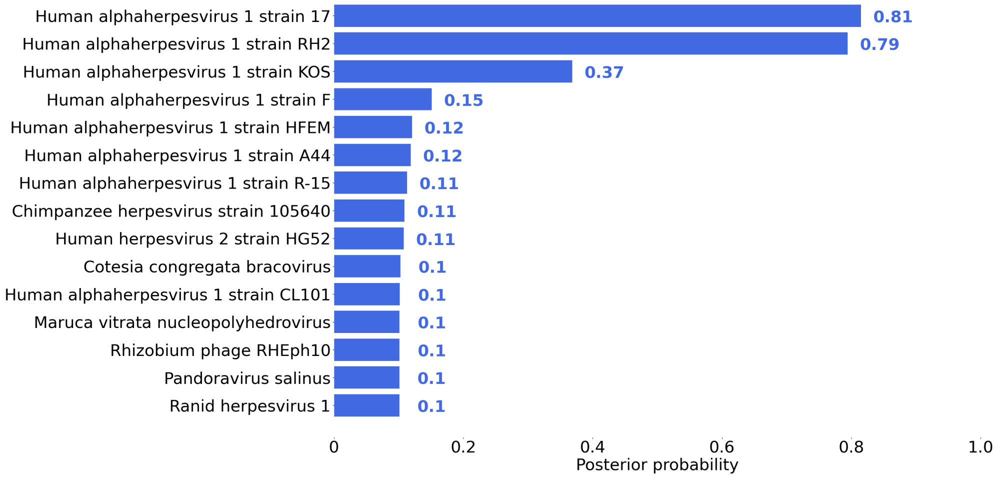

Figure 8: PepGM identification Results for the human herpesvirus sample. Bar plot representation of the 15 highest scoring taxa, with the rounded attributed score written next to each bar.

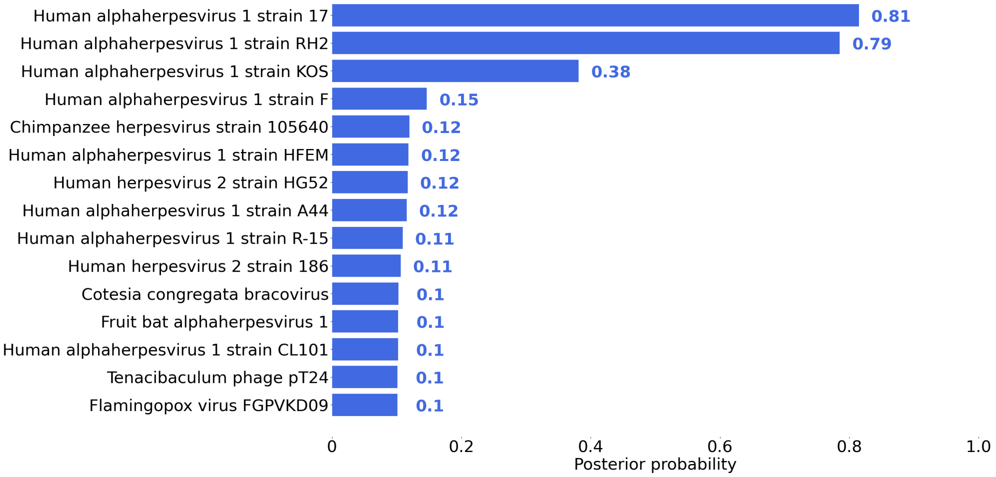

Figure 9: PepGM identification Results for the human herpesvirus sample with additional host filtering. Bar plot representation of the 15 highest scoring taxa, with the rounded attributed score written next to each bar.

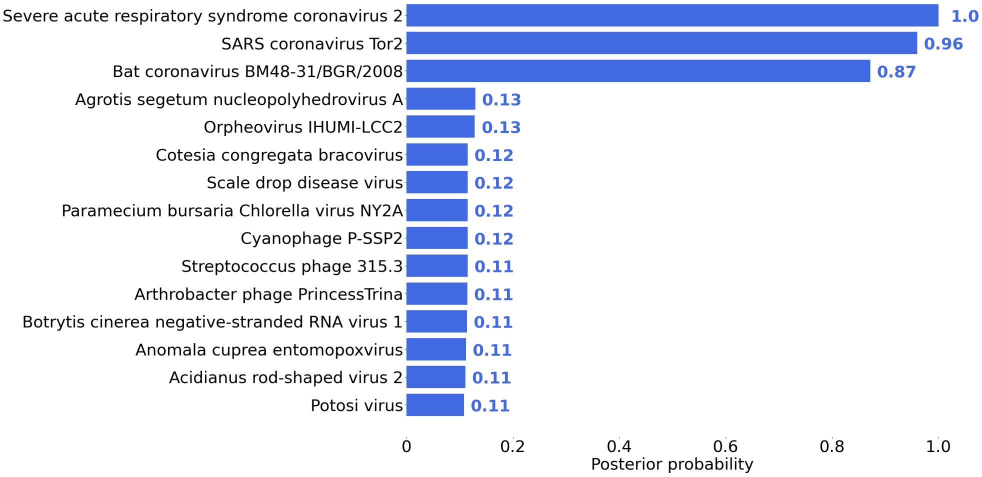

Figure 10: PepGM identification Results for the SARS-CoV-2 sample PXD025130. Bar plot representation of the 15 highest scoring taxa, with the rounded attributed score written next to each bar.

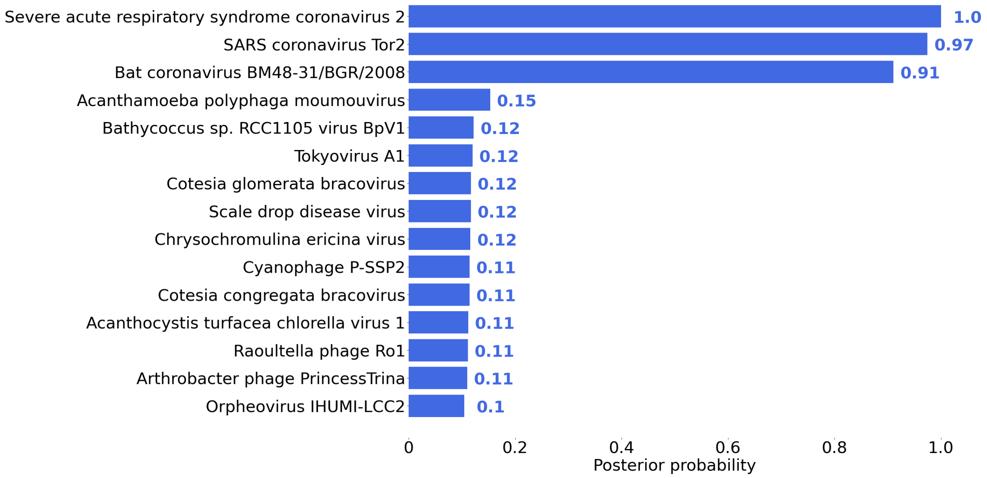

Figure 11: PepGM identification Results for the SARS-CoV-2 sample PXD025130 with additional host filtering. Bar plot representation of the 15 highest scoring taxa, with the rounded attributed score written next to each bar.

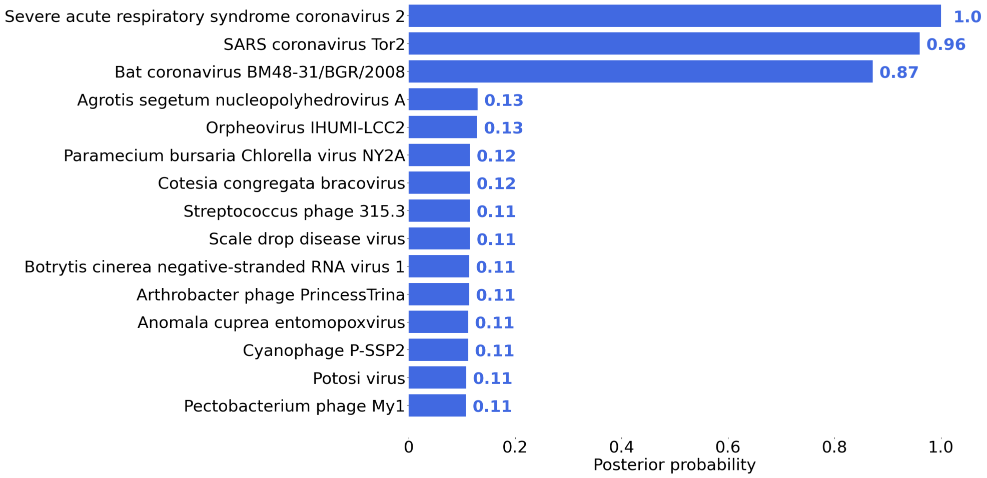

Figure 12: PepGM identification Results for the SARS-CoV-2 sample PXD025131 sample. Bar plot representation of the 15 highest scoring taxa, with the rounded attributed score written next to each bar.

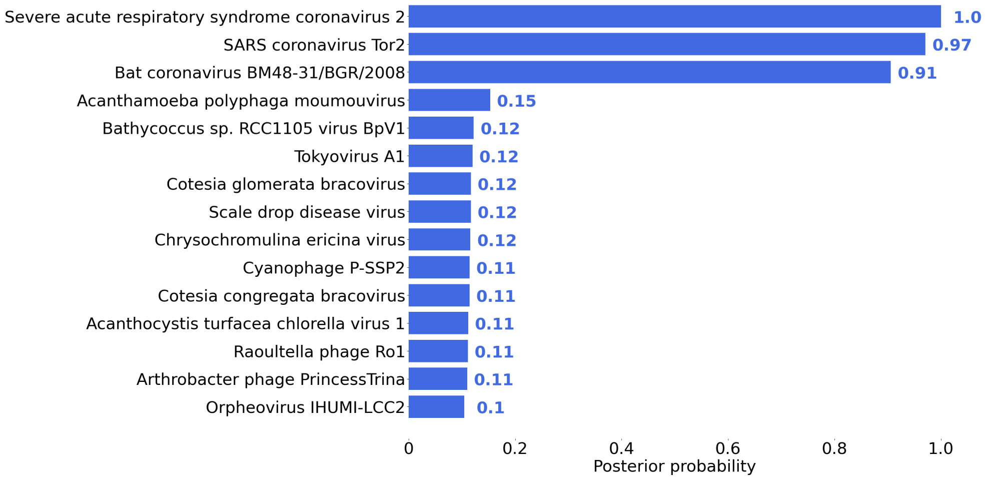

Figure 13: PepGM identification Results for the SARS-CoV-2 sample PXD025131 sample with additional host filtering. Bar plot representation of the 15 highest scoring taxa, with the rounded attributed score written next to each bar.

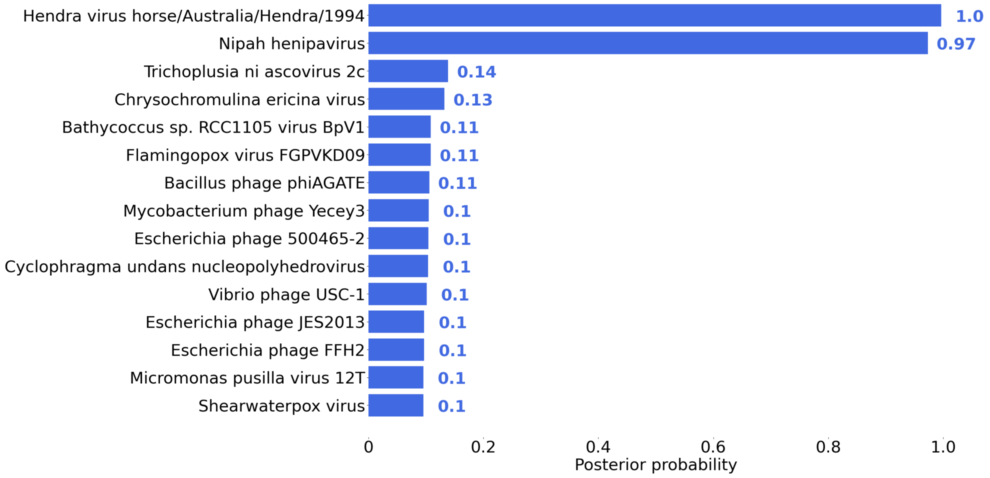

Figure 14: PepGM identification Results for the hendravirus sample. Bar plot representation of the 15 highest scoring taxa, with the rounded attributed score written next to each bar.

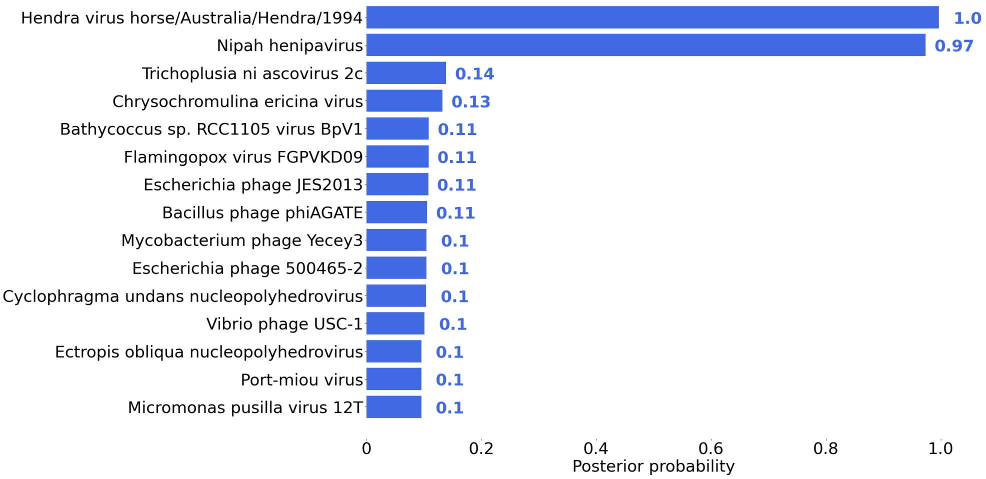

Figure 15: PepGM identification Results for the hendravirus sample with additional host filtering. Bar plot representation of the 15 highest scoring taxa, with the rounded attributed score written next to each bar.

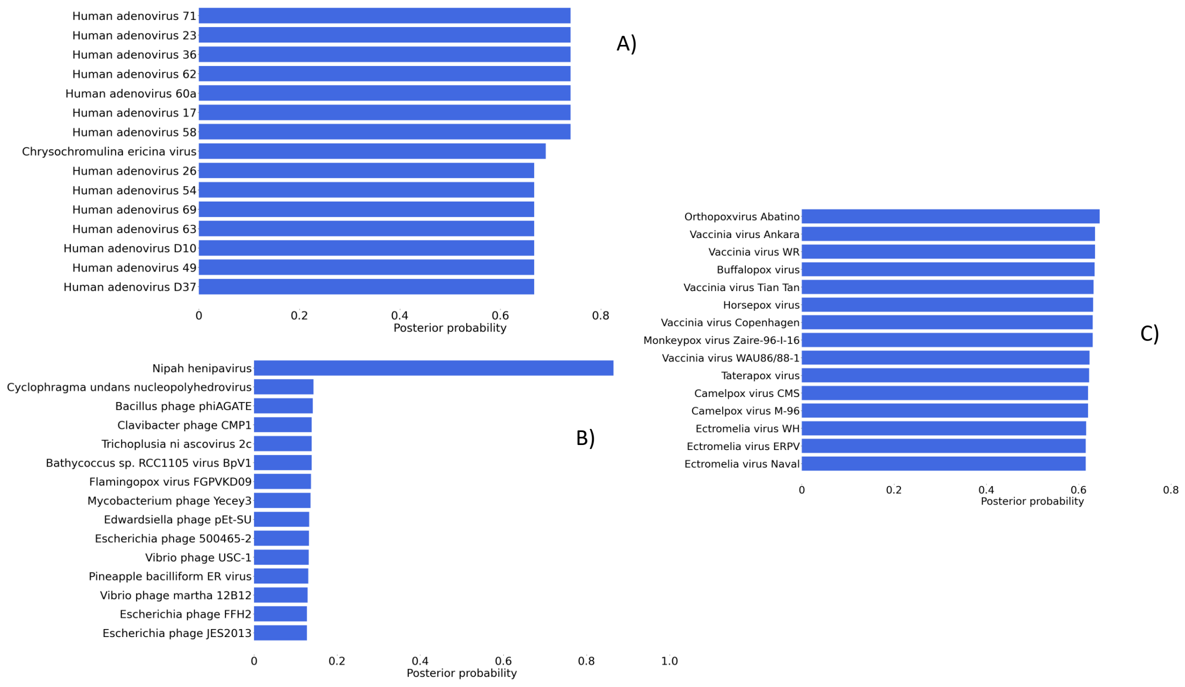

Figure 16: PepGM identification Results for A) the adenovirus sample, B) The hendravirus sample and C) the cowpox sample. For each, the correct species and/or strain (if present in the the RefSeq Viral database) was removed.

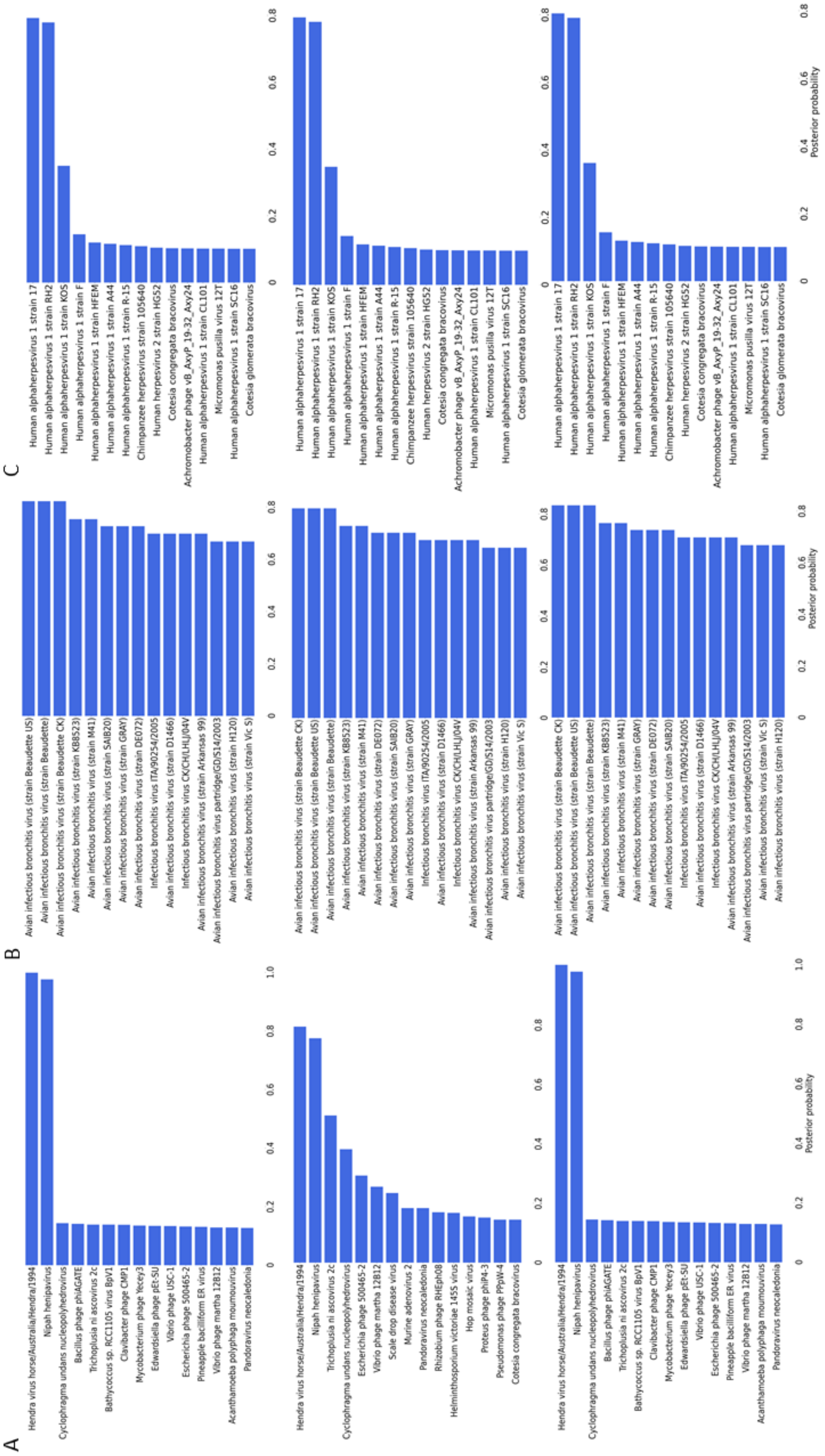

Figure 17: 2.4 PepGM results for (A) the hendravirus, (B) the avian bronchitis and (C) the herpesvirus sample. FDRs of 1%, 5% and 20% (top to bottom panels) were used for the database search step.

References

(1) Lam, S. K.; Pitrou, A.; Seibert, S. Numba: A llvm-based python jit compiler. Proceedings of the Second Workshop on the LLVM Compiler Infrastructure in HPC. 2015; pp 1–6.

(2) Huerta-Cepas, J.; Serra, F.; Bork, P. ETE 3: Reconstruction, Analysis, and Visualization of Phylogenomic Data. *Molecular Biology and Evolution* **2016**, *33*, 1635–1638.

(3) Senuma, H. mmh3. 2022; `https://github.com/hajimes/mmh3`, original-date: 2013-02-10T15:48:12Z.

(4) McKinney, W., et al. Data structures for statistical computing in python. Proceedings of the 9th Python in Science Conference. 2010; pp 51–56.

(5) Harris, C. R. et al. Array programming with NumPy. *Nature* **2020**, *585*, 357–362.

(6) Hagberg, A. A.; Schult, D. A.; Swart, P. J. Exploring Network Structure, Dynamics, and Function using NetworkX. Proceedings of the 7th Python in Science Conference. Pasadena, CA USA, 2008; pp 11 – 15.

(7) Virtanen, P. et al. SciPy 1.0: Fundamental Algorithms for Scientific Computing in Python. *Nature Methods* **2020**, *17*, 261–272.

(8) Cock, P. J. A.; Antao, T.; Chang, J. T.; Chapman, B. A.; Cox, C. J.; Dalke, A.; Friedberg, I.; Hamelryck, T.; Kauff, F.; Wilczynski, B.; de Hoon, M. J. L. Biopython: freely available Python tools for computational molecular biology and bioinformatics. *Bioinformatics* **2009**, *25*, 1422–1423.

(9) Hunter, J. D. Matplotlib: A 2D graphics environment. *Computing in Science & Engineering* **2007**, *9*, 90–95.

(10) Waskom, M. L. seaborn: statistical data visualization. *Journal of Open Source Software* **2021**, *6*, 3021.

(11) Serang, O.; Käll, L. Solution to Statistical Challenges in Proteomics Is More Statistics, Not Less. *Journal of Proteome Research* **2015**, *14*, 4099–4103, Publisher: American Chemical Society.

(12) Pearl, J. Reverend Bayes on inference engines: A distributed hierarchical approach. Cognitive Systems Laboratory, School of Engineering and Applied Science. 1982.

(13) Pearl, J. *Probabilistic Reasoning in Intelligent Systems: Networks of Plausible Inference*; Morgan Kaufmann, 1988; Google-Books-ID: AvNID7LyMusC.

(14) Kschischang, F.; Frey, B.; Loeliger, H.-A. Factor graphs and the sum-product algorithm. *IEEE Transactions on Information Theory* **2001**, *47*, 498–519.

(15) Koller, D.; Friedman, N. *Probabilistic Graphical Models: Principles and Techniques*; MIT Press, 2009; Google-Books-ID: 7dzpHCHzNQ4C.

(16) Knoll, C.; Rath, M.; Tschiatschek, S.; Pernkopf, F. Message Scheduling Methods for Belief Propagation. Machine Learning and Knowledge Discovery in Databases. Cham, 2015; pp 295–310.
